# *N*-(1-Diacetyl­amino-1*H*-tetra­zol-5-yl)acetamide

**DOI:** 10.1107/S1600536809027421

**Published:** 2009-07-18

**Authors:** Chun-Lin He, Zhi-Ming Du, Zheng-Qiang Tang, Xiao-Min Cong, Ling-Qiao Meng

**Affiliations:** a State Key Laboratory of Explosion Science and Technology, Beijing Institute of Technology, Beijing 100081, People’s Republic of China

## Abstract

In the crystal structure of the title compound, C_7_H_10_N_6_O_3_, there are N—H⋯O, N—H⋯N and C—H⋯O inter­actions, generating a three-dimensional supra­molecular network structure. A short intermolecular O⋯C contact of 2.8994 (18) Å is alsopresent in the crystal structure, but no π–π contacts are observed.

## Related literature

For the preparation, see: Gaponnik & Karavai (1984 ▶). For general background to the use of 1, 5-diaminotetrazole as an intermediate in the preparation of tetrazole-containing compounds with prospective applications in energetic mater­ials, see: Galvez-Ruiz *et al.* (2005 ▶). For hydrogen-bond-length data, see: Desiraju & Steiner (1999 ▶). For carbon­yl–carbonyl inter­actions, see: Allen *et al.* (1998 ▶).
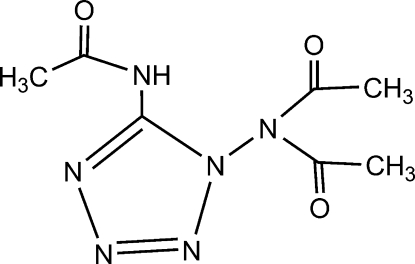


## Experimental

### 

#### Crystal data

C_7_H_10_N_6_O_3_
*M* *_r_* = 226.21
Monoclinic, 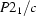

*a* = 6.973 (2) Å
*b* = 16.678 (5) Å
*c* = 8.871 (3) Å
β = 106.987 (4)°
*V* = 986.6 (5) Å^3^
*Z* = 4
Mo *K*α radiation
μ = 0.12 mm^−1^
*T* = 93 K
0.60 × 0.25 × 0.18 mm

#### Data collection

Rigaku Saturn724+ diffractometer
Absorption correction: none
7848 measured reflections
2255 independent reflections
1898 reflections with *I* > 2σ(*I*)
*R*_int_ = 0.028

#### Refinement

*R*[*F* ^2^ > 2σ(*F* ^2^)] = 0.037
*wR*(*F* ^2^) = 0.089
*S* = 1.00
2255 reflections
152 parameters
H atoms treated by a mixture of independent and constrained refinement
Δρ_max_ = 0.27 e Å^−3^
Δρ_min_ = −0.31 e Å^−3^

### 

Data collection: *CrystalClear* (Rigaku, 2008 ▶); cell refinement: *CrystalClear*; data reduction: *CrystalClear*; program(s) used to solve structure: *SHELXS97* (Sheldrick, 2008 ▶); program(s) used to refine structure: *SHELXL97* (Sheldrick, 2008 ▶); molecular graphics: *ORTEP-3* (Farrugia, 1997 ▶); software used to prepare material for publication: *WinGX* (Farrugia, 1999 ▶).

## Supplementary Material

Crystal structure: contains datablocks global, I. DOI: 10.1107/S1600536809027421/xu2550sup1.cif

Structure factors: contains datablocks I. DOI: 10.1107/S1600536809027421/xu2550Isup2.hkl

Additional supplementary materials:  crystallographic information; 3D view; checkCIF report

## Figures and Tables

**Table 1:** Hydrogen-bond geometry (Å, °)

*D*—H⋯*A*	*D*—H	H⋯*A*	*D*⋯*A*	*D*—H⋯*A*
N6—H6*N*⋯O1^i^	0.902 (17)	1.955 (17)	2.7675 (16)	149.1 (14)
N6—H6*N*⋯N1^i^	0.902 (17)	2.473 (16)	3.1359 (18)	130.6 (13)
C3—H3*A*⋯O1^ii^	0.98	2.49	3.459 (2)	169
C7—H7*C*⋯O3^iii^	0.98	2.57	3.505 (2)	159

Symmetry codes: (i) 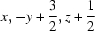; (ii) 
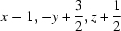
; (iii) 
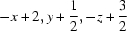
.

**Fig. 1.**
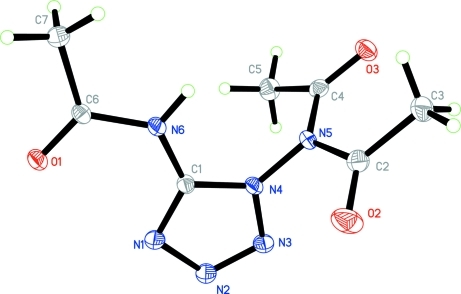
The molecular structure of the title compound with the atom-numbering scheme. Displacement ellipsoids are drawn at the 30% probability level.

## supplementary crystallographic information

### Comment

1, 5-Diaminotetrazole has been reported using as a valuable intermediate in preparation of tetrazole-containing compounds which might have prospective application in energetic materials (Gaponnik & Karavai, 1984; Galvez-Ruiz *et al.*, 2005). The presence of three acetyl groups in the title compound may put itself as an intermediate for preparing derivatives which have a bigger molecule. The title compound had been prepared by Gaponnik & Karavai (1984). Herein we report its crystal structure.

The molecular structure of the title compound is presented in Fig. 1, the bond distances and bond angles in the title compound are as expected for a molecule of this kind. The molecules are linked to each other via N—H···O, N—H···N and C—H···O hydrogen bonds (Table 1). The range for the H···O distances agree with those found for weak C—H···O hydrogen bonds (Desiraju & Steiner, 1999). The O1···C4^ii^ distance is 2.8994 (18) Å [symmetry code: (ii) x, 3/2-y, -1/2+z], this distance agrees with the disscusion of intermolecular C=O ···C=O interactions (Allen *et al.*, 1998), which may contribute to the stabilization of crystal structure.

### Experimental

The title compound was prepared according to the literature method (Gaponnik & Karavai, 1984). 220 mg of obtained product was dissolved in the mixture solution of methanol (10 ml) and acetone (20 ml) and the solution was kept at room temperature to give suitable crystals for X-ray structure determination.

### Refinement

Amino H atoms were located in a difference Fourier maps and were refined isotropically. Methyl H-atoms were placed in calculated positions with C—H = 0.98 Å, and torsion angles were refined to fit the electron density with U_iso_(H) = 1.2U_eq_(C).

### Crystal data

**Table:**

C_7_H_10_N_6_O_3_	*F*(000) = 472
*M**_r_* = 226.21	*D*_x_ = 1.523 Mg m^−^^3^
Monoclinic, *P*2_1_/*c*	Mo *K*α radiation, λ = 0.71073 Å
Hall symbol: -P 2ybc	Cell parameters from 3214 reflections
*a* = 6.973 (2) Å	θ = 3.1–27.5°
*b* = 16.678 (5) Å	µ = 0.12 mm^−^^1^
*c* = 8.871 (3) Å	*T* = 93 K
β = 106.987 (4)°	Block, colourless
*V* = 986.6 (5) Å^3^	0.60 × 0.25 × 0.18 mm
*Z* = 4	

### Data collection

**Table:**

Rigaku Saturn724+ diffractometer	1898 reflections with *I* > 2σ(*I*)
Radiation source: Rotating Anode	*R*_int_ = 0.028
graphite	θ_max_ = 27.5°, θ_min_ = 3.1°
Detector resolution: 28.5714 pixels mm^-1^	*h* = −9→8
Multi–scan	*k* = −21→20
7848 measured reflections	*l* = −11→11
2255 independent reflections	

### Refinement

**Table:**

Refinement on *F*^2^	Primary atom site location: structure-invariant direct methods
Least-squares matrix: full	Secondary atom site location: difference Fourier map
*R*[*F*^2^ > 2σ(*F*^2^)] = 0.037	Hydrogen site location: inferred from neighbouring sites
*wR*(*F*^2^) = 0.089	H atoms treated by a mixture of independent and constrained refinement
*S* = 1.00	*w* = 1/[σ^2^(*F*_o_^2^) + (0.046*P*)^2^ + 0.18*P*] where *P* = (*F*_o_^2^ + 2*F*_c_^2^)/3
2255 reflections	(Δ/σ)_max_ < 0.001
152 parameters	Δρ_max_ = 0.27 e Å^−^^3^
0 restraints	Δρ_min_ = −0.31 e Å^−^^3^

### Special details

**Table:**

Geometry. All esds (except the esd in the dihedral angle betweeex two l.s. planes) are estimated using the full covariance matrix. The cell esds are taken into account individually in the estimation of esds in distances, angles and torsion angles; correlations between esds in cell parameters are only used when they are defined by crystal symmetry. An approximate (isotropic) treatment of cell esds is used for estimating esds involving l.s. planes.
Refinement. Refinement of F^2^ against ALL reflections. The weighted R-factor wR and goodness of fit S are based on F^2^, conventional R-factors R are based on F, with F set to zero for negative F^2^. The threshold expression of F^2^ > 2sigma(F^2^) is used only for calculating R-factors(gt) etc. and is not relevant to the choice of reflections for refinement. R-factors based on F^2^ are statistically about twice as large as those based on F, and R- factors based on ALL data will be even larger.

### Fractional atomic coordinates and isotropic or equivalent isotropic displacement parameters (Å^2^)

**Table:**

	*x*	*y*	*z*	*U*_iso_*/*U*_eq_	
O1	0.98846 (13)	0.79462 (5)	0.37140 (10)	0.0163 (2)	
O2	0.33700 (14)	0.66238 (7)	0.56926 (11)	0.0280 (3)	
O3	0.77881 (14)	0.53886 (6)	0.89046 (10)	0.0199 (2)	
N1	0.71476 (16)	0.66802 (7)	0.30010 (12)	0.0170 (2)	
N2	0.60145 (16)	0.59851 (7)	0.26310 (12)	0.0182 (3)	
N3	0.56513 (16)	0.56773 (6)	0.38460 (12)	0.0170 (2)	
N4	0.65849 (16)	0.61680 (6)	0.50781 (12)	0.0141 (2)	
N5	0.64409 (15)	0.60537 (6)	0.65826 (12)	0.0137 (2)	
N6	0.85854 (15)	0.73478 (6)	0.55048 (12)	0.0147 (2)	
C1	0.74742 (18)	0.67770 (7)	0.45264 (14)	0.0133 (3)	
C2	0.46012 (19)	0.63385 (8)	0.68129 (15)	0.0176 (3)	
C3	0.4357 (2)	0.62716 (9)	0.84238 (15)	0.0216 (3)	
H3A	0.3149	0.6562	0.8459	0.026*	
H3B	0.5531	0.6504	0.9195	0.026*	
H3C	0.4232	0.5705	0.8676	0.026*	
C4	0.79059 (19)	0.55442 (7)	0.76155 (15)	0.0153 (3)	
C5	0.9522 (2)	0.52400 (8)	0.69584 (16)	0.0206 (3)	
H5A	1.0570	0.4978	0.7798	0.025*	
H5B	1.0104	0.5690	0.6532	0.025*	
H5C	0.8949	0.4853	0.6116	0.025*	
C6	0.98590 (18)	0.78772 (7)	0.50729 (14)	0.0132 (3)	
C7	1.1204 (2)	0.83460 (8)	0.63980 (15)	0.0186 (3)	
H7A	1.2488	0.8066	0.6799	0.022*	
H7B	1.0572	0.8401	0.7245	0.022*	
H7C	1.1433	0.8879	0.6019	0.022*	
H6N	0.858 (2)	0.7348 (10)	0.652 (2)	0.034 (5)*	

### Atomic displacement parameters (Å^2^)

**Table:**

	*U*^11^	*U*^22^	*U*^33^	*U*^12^	*U*^13^	*U*^23^
O1	0.0204 (5)	0.0177 (5)	0.0116 (4)	−0.0020 (4)	0.0057 (4)	0.0007 (4)
O2	0.0230 (5)	0.0397 (6)	0.0199 (5)	0.0122 (5)	0.0039 (4)	0.0046 (4)
O3	0.0220 (5)	0.0225 (5)	0.0150 (5)	0.0013 (4)	0.0051 (4)	0.0037 (4)
N1	0.0176 (6)	0.0200 (6)	0.0128 (5)	−0.0040 (4)	0.0037 (4)	−0.0021 (4)
N2	0.0191 (6)	0.0203 (6)	0.0148 (5)	−0.0039 (4)	0.0044 (4)	−0.0020 (4)
N3	0.0186 (6)	0.0177 (6)	0.0139 (5)	−0.0034 (4)	0.0034 (4)	−0.0026 (4)
N4	0.0172 (5)	0.0152 (5)	0.0099 (5)	−0.0029 (4)	0.0040 (4)	−0.0007 (4)
N5	0.0151 (5)	0.0161 (5)	0.0106 (5)	−0.0005 (4)	0.0049 (4)	0.0016 (4)
N6	0.0181 (5)	0.0169 (6)	0.0099 (5)	−0.0042 (4)	0.0051 (4)	−0.0015 (4)
C1	0.0130 (6)	0.0143 (6)	0.0130 (6)	0.0000 (5)	0.0043 (5)	0.0011 (5)
C2	0.0167 (7)	0.0192 (7)	0.0170 (6)	−0.0001 (5)	0.0051 (5)	−0.0007 (5)
C3	0.0183 (7)	0.0295 (8)	0.0187 (7)	0.0018 (5)	0.0083 (6)	−0.0004 (6)
C4	0.0152 (7)	0.0130 (6)	0.0160 (6)	−0.0024 (5)	0.0021 (5)	−0.0005 (5)
C5	0.0185 (7)	0.0217 (7)	0.0228 (7)	0.0031 (5)	0.0079 (6)	0.0018 (6)
C6	0.0142 (6)	0.0133 (6)	0.0125 (6)	0.0022 (5)	0.0045 (5)	0.0016 (5)
C7	0.0218 (7)	0.0182 (7)	0.0154 (6)	−0.0047 (5)	0.0048 (5)	−0.0027 (5)

### Geometric parameters (Å, °)

**Table:**

O1—C6	1.2164 (15)	N6—H6N	0.900 (16)
O2—C2	1.2052 (16)	C2—C3	1.4919 (18)
O3—C4	1.1987 (15)	C3—H3A	0.9800
N1—C1	1.3149 (16)	C3—H3B	0.9800
N1—N2	1.3871 (15)	C3—H3C	0.9800
N2—N3	1.2841 (15)	C4—C5	1.5004 (18)
N3—N4	1.3684 (15)	C5—H5A	0.9800
N4—C1	1.3538 (16)	C5—H5B	0.9800
N4—N5	1.3806 (14)	C5—H5C	0.9800
N5—C4	1.4346 (16)	C6—C7	1.4927 (17)
N5—C2	1.4374 (16)	C7—H7A	0.9800
N6—C1	1.3663 (16)	C7—H7B	0.9800
N6—C6	1.3834 (16)	C7—H7C	0.9800
			
C1—N1—N2	105.16 (10)	C2—C3—H3C	109.5
N3—N2—N1	111.96 (10)	H3A—C3—H3C	109.5
N2—N3—N4	105.48 (10)	H3B—C3—H3C	109.5
C1—N4—N3	108.73 (10)	O3—C4—N5	120.21 (12)
C1—N4—N5	128.57 (10)	O3—C4—C5	124.47 (12)
N3—N4—N5	122.52 (10)	N5—C4—C5	115.32 (11)
N4—N5—C4	117.38 (10)	C4—C5—H5A	109.5
N4—N5—C2	114.32 (10)	C4—C5—H5B	109.5
C4—N5—C2	127.12 (10)	H5A—C5—H5B	109.5
C1—N6—C6	124.02 (11)	C4—C5—H5C	109.5
C1—N6—H6N	117.8 (11)	H5A—C5—H5C	109.5
C6—N6—H6N	117.9 (11)	H5B—C5—H5C	109.5
N1—C1—N4	108.66 (11)	O1—C6—N6	122.10 (12)
N1—C1—N6	129.41 (11)	O1—C6—C7	122.90 (11)
N4—C1—N6	121.84 (11)	N6—C6—C7	115.00 (11)
O2—C2—N5	117.62 (12)	C6—C7—H7A	109.5
O2—C2—C3	124.49 (12)	C6—C7—H7B	109.5
N5—C2—C3	117.89 (11)	H7A—C7—H7B	109.5
C2—C3—H3A	109.5	C6—C7—H7C	109.5
C2—C3—H3B	109.5	H7A—C7—H7C	109.5
H3A—C3—H3B	109.5	H7B—C7—H7C	109.5
			
C1—N1—N2—N3	0.53 (14)	N5—N4—C1—N6	7.2 (2)
N1—N2—N3—N4	−0.89 (14)	C6—N6—C1—N1	−10.5 (2)
N2—N3—N4—C1	0.93 (13)	C6—N6—C1—N4	165.81 (11)
N2—N3—N4—N5	176.44 (11)	N4—N5—C2—O2	1.79 (17)
C1—N4—N5—C4	−96.01 (15)	C4—N5—C2—O2	−165.32 (12)
N3—N4—N5—C4	89.42 (14)	N4—N5—C2—C3	−177.37 (11)
C1—N4—N5—C2	95.54 (15)	C4—N5—C2—C3	15.51 (18)
N3—N4—N5—C2	−79.02 (14)	N4—N5—C4—O3	−175.90 (11)
N2—N1—C1—N4	0.09 (14)	C2—N5—C4—O3	−9.13 (19)
N2—N1—C1—N6	176.77 (12)	N4—N5—C4—C5	4.14 (15)
N3—N4—C1—N1	−0.63 (14)	C2—N5—C4—C5	170.91 (12)
N5—N4—C1—N1	−175.79 (11)	C1—N6—C6—O1	10.13 (19)
N3—N4—C1—N6	−177.61 (11)	C1—N6—C6—C7	−169.21 (11)

### Hydrogen-bond geometry (Å, °)

**Table:**

*D*—H···*A*	*D*—H	H···*A*	*D*···*A*	*D*—H···*A*
N6—H6N···O1^i^	0.902 (17)	1.955 (17)	2.7675 (16)	149.1 (14)
N6—H6N···N1^i^	0.902 (17)	2.473 (16)	3.1359 (18)	130.6 (13)
C3—H3A···O1^ii^	0.98	2.49	3.459 (2)	169
C7—H7C···O3^iii^	0.98	2.57	3.505 (2)	159

Symmetry codes: (i) *x*, −*y*+3/2, *z*+1/2; (ii) *x*−1, −*y*+3/2, *z*+1/2; (iii) −*x*+2, *y*+1/2, −*z*+3/2.
